# Effects of Obesity in Old Age on the Basement Membrane of Skeletal Muscle in Mice

**DOI:** 10.3390/ijms24119209

**Published:** 2023-05-24

**Authors:** Yuji Kanazawa, Yuri Ikeda-Matsuo, Hiaki Sato, Mamoru Nagano, Satoshi Koinuma, Tatsuo Takahashi, Hirokazu Suzuki, Ryo Miyachi, Yasufumi Shigeyoshi

**Affiliations:** 1 Department of Physical Therapy, Hokuriku University, Ishikawa, Kanazawa 920-1180, Japan; ry_miyachi@hokuriku-u.ac.jp; 2 Department of Anatomy and Neurobiology, Faculty of Medicine, Kindai University, Ohnohigashi, Osakasayama 589-8511, Japan; m-nagano@med.kindai.ac.jp (M.N.); koi@med.kindai.ac.jp (S.K.); shigey@med.kindai.ac.jp (Y.S.); 3 Department of Clinical Pharmacology, Hokuriku University, Ishikawa, Kanazawa 920-1181, Japan; y-matsuo@hokuriku-u.ac.jp (Y.I.-M.); t-takahashi@hokuriku-u.ac.jp (T.T.); 4 Department of Medical Technology and Clinical Engineering, Hokuriku University, Ishikawa, Kanazawa 920-1180, Japan; h-sato@hokuriku-u.ac.jp; 5 Department of Synthetic Chemistry, Hokuriku University, Ishikawa, Kanazawa 920-1181, Japan; h-suzuki@hokuriku-u.ac.jp

**Keywords:** aging, obesity, basement membrane, skeletal muscle, collagen IV

## Abstract

Obesity and aging are known to affect the skeletal muscles. Obesity in old age may result in a poor basement membrane (BM) construction response, which serves to protect the skeletal muscle, thus making the skeletal muscle more vulnerable. In this study, older and young male C57BL/6J mice were divided into two groups, each fed a high-fat or regular diet for eight weeks. A high-fat diet decreased the relative gastrocnemius muscle weight in both age groups, and obesity and aging individually result in a decline in muscle function. Immunoreactivity of collagen IV, the main component of BM, BM width, and BM-synthetic factor expression in young mice on a high-fat diet were higher than that in young mice on a regular diet, whereas such changes were minimal in obese older mice. Furthermore, the number of central nuclei fibers in obese older mice was higher than in old mice fed a regular diet and young mice fed a high-fat diet. These results suggest that obesity at a young age promotes skeletal muscle BM formation in response to weight gain. In contrast, this response is less pronounced in old age, suggesting that obesity in old age may lead to muscle fragility.

## 1. Introduction

Skeletal muscle accounts for approximately 40% of body weight and is responsible for energy production, glucose metabolism, and powering joint movement [1]. In addition, skeletal muscles have endocrine functions and participate in body weight regulation, low-grade inflammation, insulin sensitivity, tumor growth inhibition, and cognitive function [2]. Thus, maintaining skeletal muscle structure and function is essential for health maintenance. Skeletal muscle function improves in response to exercise and increases its activity through hypertrophy or increased endurance [3,4], whereas aging and obesity are known to deteriorate muscle structure and function. Aging causes muscle atrophy and loss of muscle function [5,6,7], resulting in muscle fragility [8]. Obesity in old age can also result in loss of normal muscle function as it can induce muscle wasting and decrease protein synthesis, which are associated with chronic inflammation and reduced production of anabolic hormones, respectively [9]. Furthermore, weight gain associated with obesity increases the strain on the muscle fibers. Chronic muscle overloading is expected to result in muscle hypertrophy and strengthening; however, muscle mass and strength relative to body weight are reduced by obesity [9]. Additionally, physical performance may be significantly reduced, especially in geriatric obesity, because skeletal muscles are less responsive to the overload associated with weight gain [9,10].

The basement membrane (BM) is a layer of the extracellular matrix (ECM) that covers the muscle fibers as a mechanical support structure [11]. Collagen IV, the main component of the BM, is a reticular collagen encoded by *Col4a1* and *Col4a* [11,12]. Heat shock protein 47 (HSP47) regulates the trimer formation of *Col4a1* and *Col4a2* during collagen IV syntheses, whereas matrix metalloproteinase 14 (MMP14) and MMP2 promote collagen IV degradation [13,14,15,16]. Thus, the BM is maintained by the careful regulation of both collagen IV synthesis and degradation. Collagen IV plays a crucial role in muscle integrity and its deficiency causes muscular dystrophy and muscle fiber fragility [17]. Furthermore, the induction of synthetic factors such as *Col4a1* and *Hsp47* has been reported to be critical for the recovery process of disuse muscle atrophy [8]. Additionally, muscle injury recovery requires the induction of not only synthetic factors but also degradation factors, indicating the occurrence of BM remodeling [18]. These facts suggest that the BM is important not only for protecting muscles from mechanical stimuli, but also for muscle recovery. Therefore, for skeletal muscle to be protected from mechanical stimuli and to maintain structure and function, the continuous reconstruction of the BM through maintaining the balance between synthesis and degradation of collagen IV is essential.

Previous studies reported that skeletal muscle BM construction is promoted during muscle recovery and habitual exercise [18,19] but conversely inhibited by aging [20]. Interestingly, it has been reported that obese mice may have impaired ECM recovery after acute muscle injury compared to control mice [21]. Thus, obesity is expected to have an adverse effect on BM construction; however, the impact of obesity on the skeletal muscle BM structure and the expression of BM-related factors remains unclear. Furthermore, the impact of combined obesity and aging on BM construction remains unresolved. This study investigated the effects of obesity on skeletal muscle BM construction and structure in older mice. We observed morphological and molecular changes in myofibers and the BM in the gastrocnemius muscles of young and aged mice due to high-fat diet-induced obesity.

## 2. Results

### 2.1. Dietary Intake, Body Weight, and Visceral Fat Content

**Figure 1:**
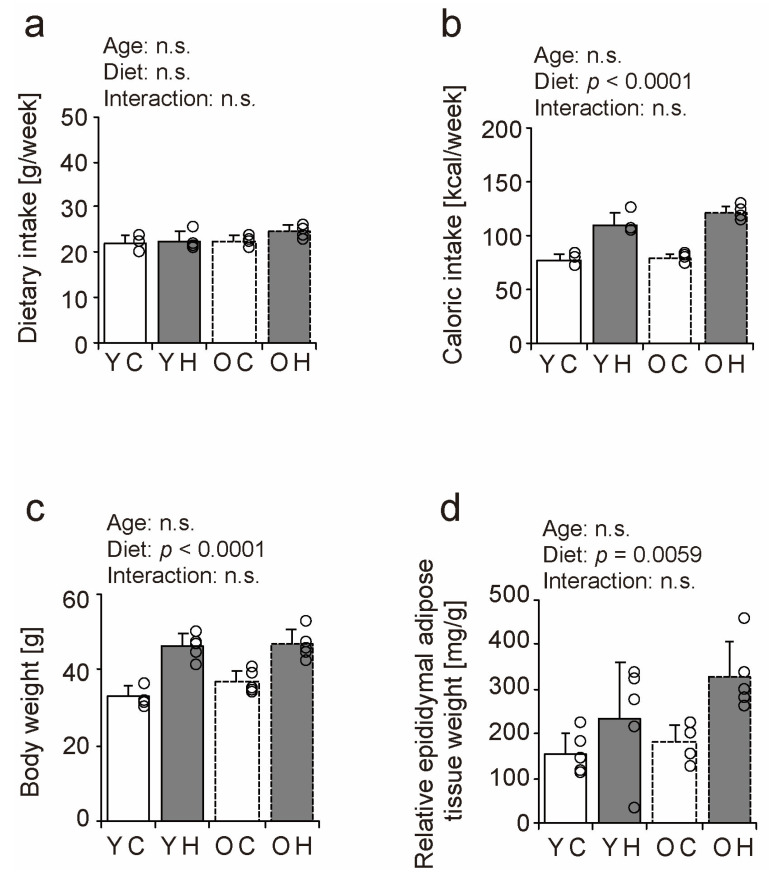
Comparison of dietary intake, body weight, and visceral fat content. Weekly dietary intake (**a**) and caloric intake (**b**). At the end of the experimental period, body weight (**c**) and epididymal fat mass as a measure of visceral fat were measured and calculated relative to body weight (**d**). Data are presented as the mean ± standard deviation, *n* = 4~5 per group. YC, young mice + control diet; YH, young mice + high-fat diet; OC, older mice + control diet; OH, older mice + high-fat diet group. n.s.: not significant.

To confirm the association of obesity with an eight-week high-fat diet in young and older mice, weekly dietary intake, weekly caloric intake, body weight, and relative epididymal adipose tissue weight were measured as a measure of visceral fat. Although mean dietary intake during the experimental period did not differ significantly by age or diet type (Figure 1a), caloric intake per unit in the high-fat diet was approximately 1.4-fold higher than that in the control diet (Supplementary Table S1), and caloric intake increased under the influence of the high-fat diet (Figure 1b). Body weight (Figure 1c) and relative epididymal adipose tissue weight (Figure 1d) increased with the consumption of the high-fat diet in both age groups. The present study confirmed that young and older mice became obese after eight weeks of high-fat diet intake.

### 2.2. Relative Muscle Weight and Fiber Cross-Sectional Area

**Figure 2:**
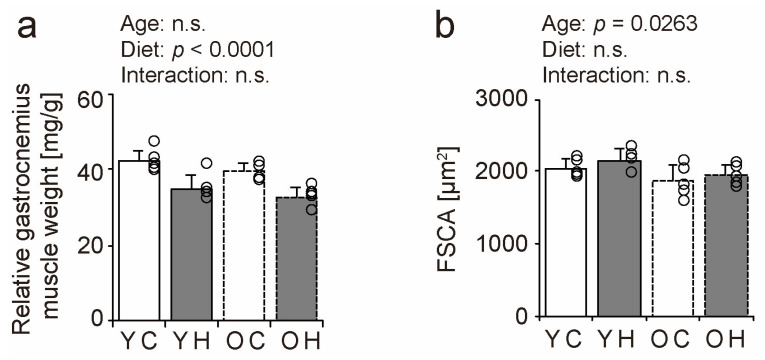
Comparison of relative muscle weight and fiber cross-sectional area. Relative muscle mass (**a**) and fiber cross-sectional area (FCSA) (**b**) of the gastrocnemius muscle is shown. Data are presented as the mean ± standard deviation, *n* = 5 per group. YC, young mice + control diet; YH, young mice + high-fat diet; OC, older mice + control diet; OH, older mice + high-fat diet group. n.s.: not significant.

To determine the effect of obesity and aging on skeletal muscles, we measured the relative gastrocnemius muscle weight and fiber cross-sectional area (FCSA) in the young mice + control diet (YC), young mice + high-fat diet (YH), older mice + control diet (OC), and older mice + high-fat diet (OH) groups (Figure 2). The relative gastrocnemius muscle weight decreased in both older and young mice owing to weight gain from the high-fat diet (Figure 2a). The ratio of the decrease in the relative muscle weight was comparable between the young (7.1%) and the older mice (6.9%). The FCSA of the gastrocnemius muscle decreased significantly with aging but was not affected by a high-fat diet, and there was no interaction between aging and a high-fat diet (Figure 2b). These results suggest that obesity and aging are negative factors for skeletal muscle integrity and result in a decrease in relative muscle weight or FCSA.

### 2.3. Muscle Function

**Figure 3:**
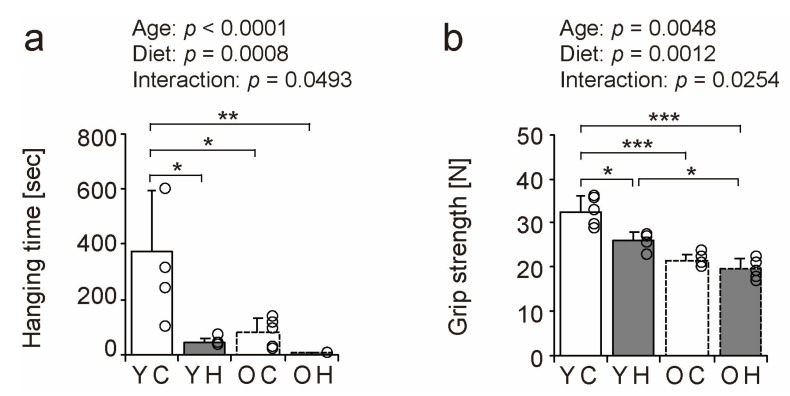
Comparison of muscle function. To assess muscle function, a wire-hanging test with the four limbs (**a**) and the grip strength of the four limbs were measured (**b**). Data are presented as the mean ± standard deviation, *n* = 5 per group. YC, young mice + control diet; YH, young mice + high-fat diet; OC, older mice + control diet; OH, older mice + high-fat diet group. *** *p <* 0.0001, ** *p <* 0.001, * *p <* 0.05.

We performed wire-hanging and grip strength tests in the YC, YH, OC, and OH groups to determine the effect of obesity and aging on muscle function (Figure 3). Hanging time and grip strength declined due to aging and high-fat diet intake (Figure 3a,b). The hanging time was significantly shorter in the YH (*p* = 0.0018), OC (*p* = 0.0046), and OH (*p* = 0.0006) groups than in the YC group (Figure 3a). The grip strengths of the YH (*p* = 0.0022), OC (*p* < 0.0001), and OH (*p* < 0.0001) groups were significantly lower than those of the YC group, and the grip strength of the OH group (*p* = 0.0025) was considerably lower than that of the YH group (Figure 3b). These results suggest that obesity and aging individually result in a decline in muscle function.

### 2.4. Histopathologic Findings

**Figure 4:**
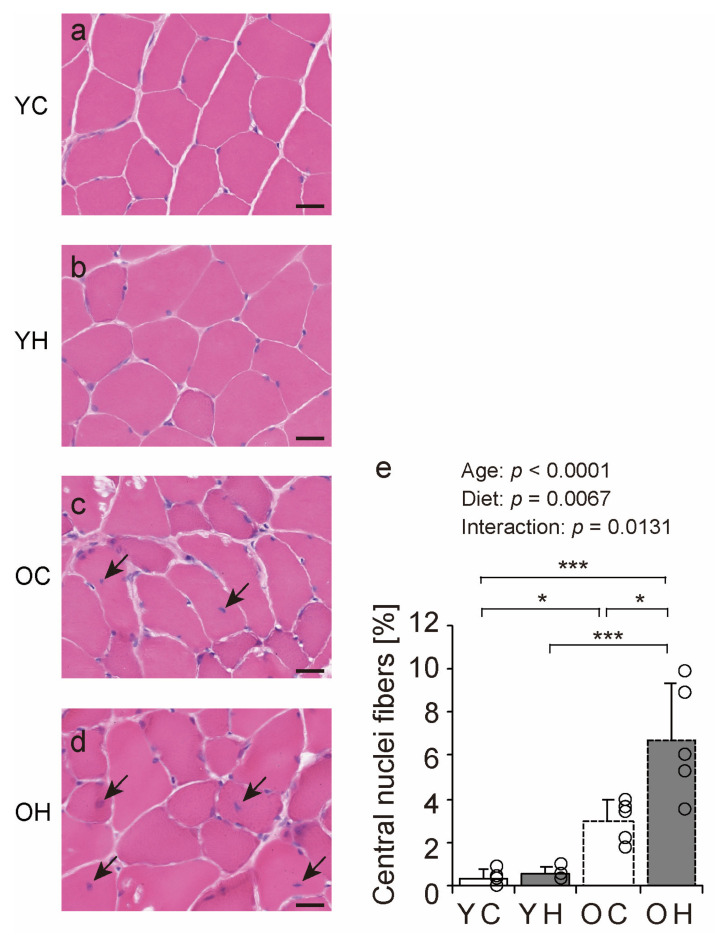
Hematoxylin and eosin staining of gastrocnemius muscles and histopathologic findings. Cross-sections of the gastrocnemius muscle were stained by hematoxylin and eosin in the YC (**a**), YH (**b**), OC (**c**), and OH (**d**) groups. Arrowheads indicate central nuclei fiber. The scale bar represents 25 µm. The number of central nuclei fiber (**e**) was measured in the images. Data are presented as the mean ± standard deviation, *n* = 5 per group. YC, young mice + control diet; YH, young mice + high-fat diet; OC, older mice + control diet; OH, older mice + high-fat diet group. *** *p <* 0.0001, * *p <* 0.05.

We examined hematoxylin and eosin-stained transverse sections of the lateral head of the gastrocnemius muscle to investigate the effects of obesity on muscle fibers in old age (Figure 4a–d). Histopathologic observations of muscle cross-sections revealed a trend toward an increase in the number of central nuclei fibers with aging and a high-fat diet (Figure 4, arrowheads in c and d). The number of central nuclei fibers was significantly higher in the OH than in the YC (*p* < 0.0001), the YH (*p* < 0.0001), and the OC (*p* = 0.0036) (Figure 4e). Central nuclei fibers appear when muscle fibers become fragile owing to aging, myopathy, and muscle regeneration [22,23,24]. Thus, obesity can increase the number of central nuclei fibers in old age.

### 2.5. Collagen IV Localization

**Figure 5:**
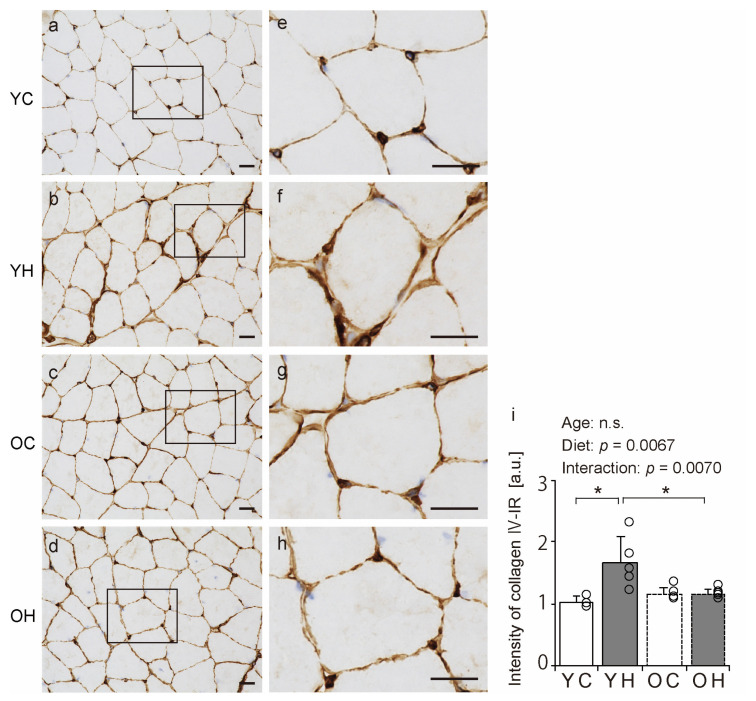
Comparison of collagen IV localization. Cross-sections of the gastrocnemius muscle were stained by an anti-collagen IV antibody. Staining images of the YC (**a**,**e**), YH (**b**,**f**), OC (**c**,**g**), and OH (**d**,**h**) are shown. (**e**–**h**) are enlargements of the rectangular regions in (**a**–**d**). The scale bar represents 25 µm. Collagen IV-immunoreactivity (IR) intensity was measured in the images (**i**). Data are presented as the mean ± standard deviation, *n* = 5 per group. YC, young mice + control diet; YH, young mice + high-fat diet; OC, older mice + control diet; OH, older mice + high-fat diet group. * *p <* 0.05. n.s.: not significant.

Collagen IV is a significant component of the BM [11]. Therefore, we assessed the BM structure using immunohistochemical analysis for collagen IV detection. In all the groups, the muscle fibers’ collagen IV immunoreactivity (IR) confirmed the presence of the BM (Figure 5a–h). Additionally, the collagen IV-IR intensity was measured. There was an interaction between aging and high-fat diet intake (Figure 5i); collagen IV-IR intensity in the YH was significantly higher than in the YC (*p* = 0.0023) and OH (*p* = 0.015). These results suggest that a high-fat diet at a young age increases collagen IV-IR intensity, whereas this response is less likely in old age.

### 2.6. Electron Microscopy Analysis

**Figure 6:**
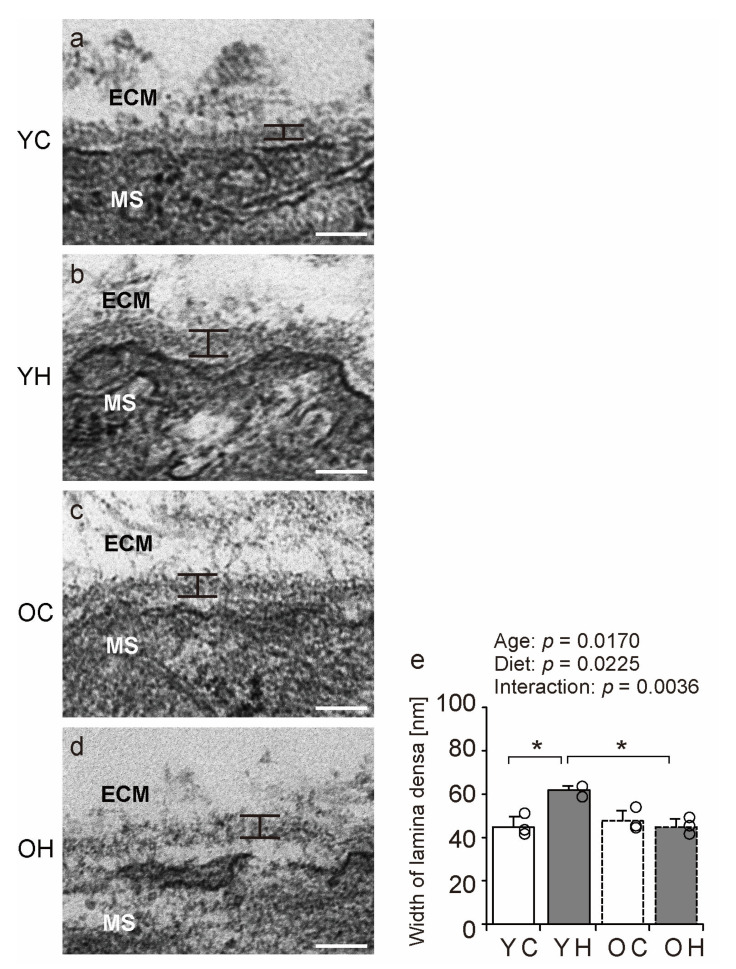
Electron microscopy analysis. Longitudinal ultrathin sections of gastrocnemius muscle from the YC (**a**), YH (**b**), OC (**c**), and OH (**d**) groups were examined using transmission electron microscopy. MS: muscle fibers, ECM: extracellular matrix. Scale bar = 100 nm. Lamina densa width (**e**) was measured using stained images. Data are presented as mean ± standard deviation. Each group *n* = 3. YC, young mice + control diet; YH, young mice + high-fat diet; OC, older mice + control diet; OH, older mice + high-fat diet group. * *p <* 0.05.

Electron microscopy was performed to evaluate the structure of the BM in detail. Notably, the width of the lamina densa of the YH was significantly greater than that of the YC (*p* = 0.0055) and OH (*p* = 0.0047) (Figure 6). However, the width of muscle fibers did not differ significantly between the groups (Supplementary Table S2). These results suggest that obesity in adolescence induces only BM-specific structural changes in the gastrocnemius, whereas such changes are rare in old age.

### 2.7. BM-Related Factors

**Figure 7:**
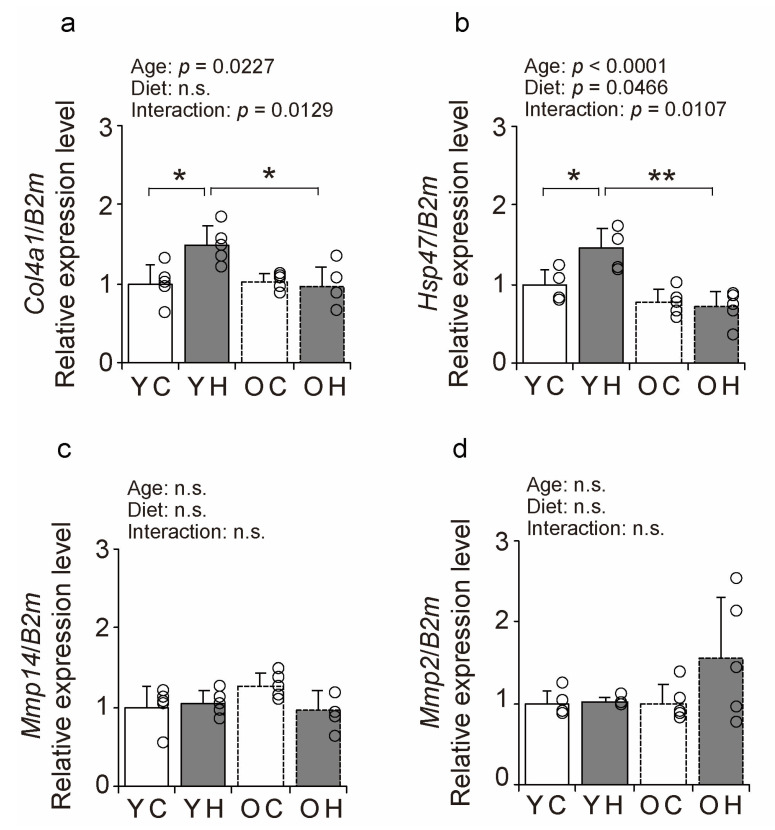
Comparison of BM-related factors expression. Changes in relative mRNA expression levels of *Col4a1* (**a**), *Hsp47* (**b**), *Mmp14* (**c**), and *Mmp2* (**d**) in the YC, YH, OC, and OH groups. Data are presented as the mean ± standard deviation, *n* = 5 per group. YC, young mice + control diet; YH, young mice + high-fat diet; OC, older mice + control diet; OH, older mice + high-fat diet group. ** *p <* 0.001, * *p <* 0.05. n.s.: not significant.

The expression of BM-related factors was measured using a quantitative polymerase chain reaction (qPCR) (Figure 7a–d) to elucidate the molecular mechanisms of the changes in collagen IV-IR intensity and BM structure. The expression of the synthetic marker *Col4a1* was significantly increased in the YH compared to that in the YC (*p* = 0.0151), and OH (*p* = 0.0084) (Figure 7a). The expression of the synthetic marker *Hsp47* was also significantly increased in the YH compared to that in the YC (*p* = 0.0124) and OH (*p* = 0.0001) (Figure 7b). There were no significant differences in the expression of the degradation markers *Mmp14* and *Mmp2* between the groups (Figure 7c,d). These results suggest that high-fat diet intake at a young age promotes collagen IV synthesis at the transcriptional level but that such responsiveness is lacking in old age.

## 3. Discussion

In the present study, we investigated the effects of obesity in old age on the BM of skeletal muscles. The significant findings of the present study were as follows: (1) the number of central nuclei fibers was increased in obese mice of old age; (2) the collagen IV-IR intensity was increased by obesity in the young but not by obesity in old age; (3) the width of the lamina densa was increased by obesity in the young, but not by obesity in old age; and (4) collagen IV synthetic factor was increased by obesity in the young, but not by obesity in old age. These findings suggest that the BM construction response is poor in geriatric obesity and skeletal muscle may be fragile.

In the present study, the number of central nuclei fibers increased with obesity in old age. As central nuclei fibers appear when muscle fibers become fragile with aging and myopathies [22,23,24], obesity in old age may result in skeletal muscle fragility. Alterations in the factors involved in the organization and integration of the plasma membrane and the BM, which are responsible for protecting muscle fibers [11], are also known to weaken the muscle and cause the appearance of central nuclei fibers [25,26]. Furthermore, the age-related decrease in collagen IV expression increases the number of damaged muscle fibers and central nuclei fibers during recovery after disuse muscle atrophy [8]. These findings suggest that obesity in old age may suppress the protective function of muscle fibers, leading to muscle fragility.

This study showed that collagen IV, a BM component of skeletal muscle, is affected by obesity and that the effect of obesity on the BM component varies with age, as collagen IV-IR intensity is increased in young obese mice but not in obese mice of old age. The IR of collagen IV increases with exercise-induced BM construction [19] and decreases with stretching-induced BM degradation [27], and thus is a potent indicator for reflecting the changes in BM construction and structure in skeletal muscle. Therefore, the results of this study suggest that obesity may promote BM construction in the gastrocnemius muscle and that aging may inhibit these changes. The IR of collagen VI and collagen IV localized in the BM [28] was also increased due to obesity in young mice but did not change significantly in older mice (Supplementary Figure S2). These results support the theory that changes in the BM components occur due to obesity. Other studies in middle-aged adults have reported that obesity can increase collagen IV-IR intensity in the adipose tissue and alter BM composition [29]. In addition, studies on mice have reported that collagen I levels in the adipose tissue increase with obesity [30]. The present findings and previous studies [29,30] indicate that obesity at a young age promotes collagen production in the skeletal muscle and adipose tissue. Furthermore, a previous study reported that collagen production by fibroblasts is suppressed with aging [31]. Thus, the responsiveness of collagen production within the skeletal muscle may be impaired during obesity in old age.

In the present study, transmission electron microscopy observations revealed that the lamina densa width of the YH was significantly increased compared to that of the YC and OH. However, in older mice, the lamina densa of the OH group did not show a significant difference. These findings suggest that older mice lose the ability to strengthen the BM in response to the load caused by obesity. Collagen IV is one of the major components of the lamina densa in the BM [11,32]. The increase in collagen IV-IR in the gastrocnemius muscle, as shown in the present study, can indicate that the increase in collagen IV is caused by an increase in the lamina densa width induced by obesity. A previous study demonstrated that collagen IV protects muscle fibers [11]. Furthermore, increased collagen IV levels have been reported to increase BM strength [33,34]. Thus, an increase in the width of the lamina densa induced by obesity is an adaptive response of the BM. This adaptive response to weight-bearing after disuse muscle atrophy was blunted by aging in our previous study as well [8]. In conjugation with these findings, the present study suggests that adaptive responses to body weight loading are retarded in older mice with respect to changes in the BM structure.

The expression of collagen IV synthetic factor in the present study was increased in the YH but not in the OH. This finding suggests that the BM production response after weight-loading is hampered at the molecular level in older mice. Contrastingly, a previous study reported that exercise loading in the elderly increases the gene expression of ECM-related factors in skeletal muscle [35]. In our previous studies, the expression of BM-related factors was also induced by the endurance habit in older rats [19]. These contradictory findings suggest that the processes underlying skeletal muscle BM production remain responsive to exercise at the molecular level, even in old age. Thus, this raises questions on what does account for the different levels of responsiveness during exercise and in obesity in old age at a molecular level. Collagen production by fibroblasts has been reported to be responsive to mechanical stress and is more enhanced under stress [36]. However, collagen gene induction is reduced in aged fibroblasts [37]. The mechanical stress on muscle associated with weight gain is minor compared to exercise and may not have been an effective stimulus for collagen induction in aging fibroblasts.

The findings of the present study are accompanied by several limitations. First, this study used only a 60% fat diet, which is capable of rapidly inducing obesity. This type of fat diet is known to distort the fat content of mice to a greater extent than a normal diet and elicits a more exaggerated metabolic response [38]. To determine the effect of dietary fat content on skeletal muscle, comparisons should be made with diets of different fat content. Second, this study did not examine the relationship between changes in the BM structure and insulin resistance. A previous study reported that obesity can promote insulin resistance by depositing the ECM in adipose tissue [39]. An analysis of glucose metabolism is needed to investigate the effects of changes in the BM structure of skeletal muscle on insulin resistance. Third, the present study analyzed the gastrocnemius muscle, a fast-twitch muscle that is susceptible to aging. Previous studies reported that collagen IV content differs between slow- and fast-twitch muscles [40]. Furthermore, unlike the present study, previous studies reported that collagen accumulation occurs with aging, and that discrepancies between different studies may be related to differences in the types of muscle fibers analyzed and the experimental techniques [40,41,42]. Therefore, future studies should examine the effects of aging and obesity on the BM structure by muscle fiber type and experimental technique. Fourth, in this study, the only element of data suggestive of muscle fragility was the measurement of the number of central nuclei fibers. To strengthen the evidence of fragility, it is necessary to assess the susceptibility of muscle to damage for the same load using a model of muscle damage caused by electrical stimulation [43]. Finally, collagen IV-IR intensity, lamina densa width, and collagen IV synthetic factor were increased in the YH but not in the OH. This reveals the influence of aging on the responsiveness to obesity; however, the exacerbating effects of aging and obesity on the skeletal muscle BM remain unexplained. More evidence is needed to understand the detailed mechanisms by which aging and obesity affect the skeletal muscle BM.

## 4. Materials and Methods

### 4.1. Animals

A cohort of 80-week-old and 9-week-old C57BL/6J male mice (*n* = 20) was obtained from the Jackson Laboratory (Kanagawa, Japan). For one month prior to the start of the experiment, the animals were raised on the same regular diet, Charles River Formula-1 diet (Oriental Yeast, Tokyo, Japan). The dietary intake and body weight before the start of the experiment are shown in Supplementary Figure S1. After one month of rearing, 13-week-old male mice (*n* = 10) were used as young mice and 84-week-old male mice (*n* = 10) were used as older mice.

All the animals were housed in individual cages and allowed free access to food and water. The environmental conditions were maintained at 23 ± 2 °C and a 12:12 h light:dark cycle. This study was approved by the Committee of Animal Care and Use of the Hokuriku University (approval number is 22-13; approval date is 3 March 2022). All experimental procedures were conducted following institutional guidelines for using experimental animals.

### 4.2. Diet 

Two diets were used in this study: AIN-93M as the control diet and HFD-60 as the high-fat diet (Oriental Yeast, Tokyo, Japan). The nutritional compositions of the diets are presented in Supplementary Table S1. Dietary and caloric intake was measured weekly during the experimental period. 

### 4.3. Group 

Young and older mice (*n* = 10 each) were divided into four groups (*n* = 5 per group): YC, YH, OC, and OH.

### 4.4. Wire Hanging Test and Grip Strength Test

At the end of the experimental period, a wire hanging test with four limbs and a grip strength test were performed to evaluate muscle function. In the wire-hanging test, the mouse was hung on the wire mesh with four limbs, and the time until the mouse fell to the soft and safe floor was measured as the hanging time, according to a previous study [44]. The hanging time was measured three times for each mouse. The interval was 1 min, and the maximum hanging time was 600 s. According to the manufacturer’s instructions, a grip strength test with all four limbs was performed using a grip strength meter (GPM-101B; Melquest, Toyama, Japan). Each mouse was placed on a metal grid attached to a grip strength meter and gently pulled from the base of the tail until release. Peak grip strength (N) was measured three times for each mouse. The time intervals were 1 min. The mean value of three trials was used to statistically analyze the wire hanging and grip strength tests.

### 4.5. Sampling 

At the end of the experimental period, the mice were weighed and anesthetized by cervical dislocation, and the gastrocnemius muscles and epididymal adipose tissue were removed and weighed. The right and left gastrocnemius muscles were used for molecular biological and morphological analyses, respectively. Part of the right gastrocnemius muscle belly was excised and preserved in RNAlater (Thermo Fisher Scientific, Waltham, MA, USA), and other parts of the muscles were frozen immediately in isopentane, cooled in dry ice, and stored at −80 °C for further analyses. 

### 4.6. Quantitative Polymerase Chain Reaction 

Total RNA was extracted from the gastrocnemius muscle using TRIzol™ reagent (Thermo Fisher Scientific, Waltham, MA, USA). After confirming its quality, the total RNA concentration was normalized to 0.5 μg. First-strand cDNA was synthesized using random primers and ReverTra Ace (Toyobo, Osaka, Japan). TB Green Premix Ex Taq II (Takara Bio, Shiga, Japan) and CFX96 Touch Real-Time PCR Detection System (Bio-Rad Laboratories, Hercules, CA, USA) were used for qPCR assays with the following thermocycling parameters: one cycle of 95 °C for 30 s followed by 40 cycles of 95 °C for 5 s and 60 °C for 30 s. A calibration curve was created using the template, and each target gene’s amount was measured. The expression level of each target gene was normalized to that of the housekeeping gene beta-2-microglobulin. The upregulation or downregulation of the target gene was calculated as the rate of altered expression compared to that of the YC. The primers used for the qPCR were as follows: 

*Col4a1*, 5′-ATGCCAGGAAGAGCAGGAAC-3′ (Forward) and 5′-CGACTACCAGGAAAGCCAACTC-3′ (Reverse); 

*Hsp47*, 5′-CGCAGCAGTAAGCAACACTACA-3′ (Forward) and 5′-TCCACATCCTTGGTGACCTCT-3′ (Reverse);

*Mmp14*, 5′-GGATACCCACTTTGATTCTGCTG-3′ (Forward) and 5′-GGAGGGGTCGTTGGAATGT-3′ (Reverse);

*Mmp2*, 5′-AAGAAAATGGACCCCGGTTT-3′ (Forward) and 5′-CAACTTCAGGTAATAAGCACCCTTG-3′ (Reverse);

*B2m*, 5′-TTCTGGTGCTTGTCTCACTGA-3′ (Forward) and 5′-CAGTATGTTCGGCTTCCCATTC-3′ (Reverse).

### 4.7. Histochemical Analyses

Transverse tissue sections (10-µm thickness) were cut from the middle part of the lateral head of the gastrocnemius muscle belly using a cryostat (CM1950; Leica, Wetzlar, Germany) at −25 °C and were mounted onto amino silane-coated glass slides. Sections were stained with hematoxylin and eosin. 

### 4.8. Immunohistochemical Analysis 

Transverse sections (10-µm thickness) were fixed in 4% paraformaldehyde and were rinsed with phosphate-buffered saline (PBS) (pH 7.4). The sections were then bleached with 3% H_2_O_2_, rinsed with PBS, and incubated for 1 h at 4 °C in PBS containing 1% normal goat serum and 0.3% Triton X-100. The sections were then incubated for 24 h at 4 °C in rabbit polyclonal anti-collagen IV antibody (ab6586; Abcam, Cambridge, MA, USA) diluted 1:500 in PBS containing 0.3% Triton X-100. The sections were subsequently incubated for 1 h at room temperature in biotinylated anti-rabbit immunoglobulin G (Vectastain ABC kit; Vector Laboratories, Berlingame, CA, USA) diluted 1:1000 in PBS, after which they were incubated in avidin-biotin complex (Vectastain ABC kit) for 1 h at 4 °C. After rinsing with PBS, the sections were washed with Tris-HCl buffer (pH 7.4) and incubated with diaminobenzidine (0.035%) in Tris-HCl buffer (0.001% H_2_O_2_) for 15 min at room temperature. After the diaminobenzidine reaction, the sections were stained with hematoxylin, dehydrated using a graded series of ethanol rinses, immersed in xylene, and embedded in Permount™ Mounting Medium (Falma Inc., Tokyo, Japan).

### 4.9. Electron Microscopy 

Longitudinal tissue sections (1 mm thickness) were cut from the middle part of the lateral gastrocnemius muscle belly at −25 °C. The sections were fixed with 4% paraformaldehyde/2% glutaraldehyde and then treated using osmium tetroxide, after which they were dehydrated using a series of ethanol gradients [8]. Finally, the sections were embedded in Epon. Ultrathin sections (90-nm thickness) were cut using an ultra-microtome and stained with 4% uranyl acetate and 1% lead citrate. Sections were analyzed using a transmission electron microscope (HT7700; Hitachi, Tokyo, Japan). 

### 4.10. Morphological Analysis

The FCSA was measured by immunohistochemical staining with an anti-collagen IV antibody. The area per image was 393,880 μm^2^, and three images were taken randomly (BZ-X700; Keyence, Osaka, Japan), and >300 muscle fibers were analyzed per mouse. Semi-quantitative analysis was performed to detect the intensity of collagen IV-IR [45]. The area per image was 393,880 μm^2,^ and three images were analyzed using ImageJ Fiji [46]. For electron microscopy, 20 images per sample were randomly obtained, and lamina densa was measured using ImageJ Fiji [46], with a reference area of 84.5 μm^2^. The width at each site was randomly measured at three points per image. The BM comprises the lamina fibroreticularis, lamina densa, and lamina lucida [32]. We measured the width of the lamina densa, which is always easily observed and has a high electron density.

### 4.11. Statistical Analysis 

KaleidaGraph statistical analysis software version 4.5.1 (Synergy Software, Reading, PA, USA) was used for statistical analysis. A two-way analysis of variance was used to test the interaction between aging and a high-fat diet. If the interaction was significant, Tukey’s honestly significant difference test was used as a post hoc test to compare all groups. A *p*-value of <0.05 was considered statistically significant. All data are expressed as means ± standard deviation.

## 5. Conclusions

In summary, we found that obesity at a young age increases collagen IV-IR and lamina densa width and upregulates *Col4a1* and *Hsp47* expression, suggesting that obesity may be a stimulus for inducing BM production. In contrast, no such reactivity was observed in obese individuals of older age, and the number of central nuclei fibers markedly increased; this may indicate that muscle structure is more fragile in older obese individuals.

The present study did not analyze the detailed effects of obesity-induced BM construction and age-related suppression of BM construction on muscle function. Further investigation is required to support these findings.

## Data Availability

All data are included in the manuscript.

## Supplementary Materials

The following supporting information can be downloaded at: https://www.mdpi.com/article/10.3390/ijms24119209/s1.
