# Supplementary material for: Effects of Obesity in Old Age on the Basement Membrane of Skeletal Muscle in Mice
Source: Int J Mol Sci. 2023 May 24;24(11):9209. doi: 10.3390/ijms24119209 (PMC10252234; doi:10.3390/ijms24119209)
Supplement: Supplementary file 1 [file ijms-24-09209-s001.zip › ijms-2403898-supplementary.pdf]

Supplementary data

1. Materials and Methods

1.1. Dietary intake and body weight

Dietary intake and body weight prior to the start of the experiment were as follows.

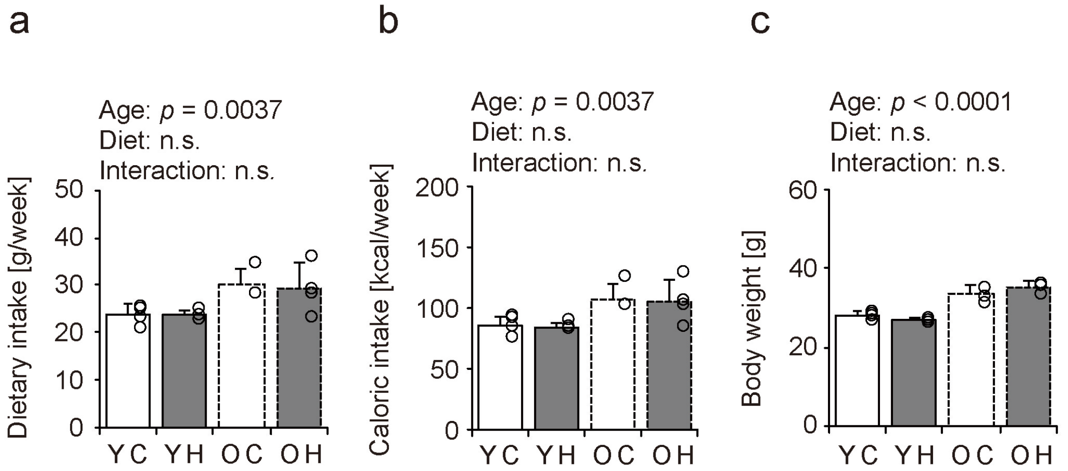

Figure S1. Comparison of dietary intake and body weight. Weekly dietary intake (a), caloric intake (b), and body weight (c) of thirteen-week-old male mice as young mice and eighty-four-week-old male mice as older mice. Data are presented as mean ± standard deviation, n = 4 per group. YC, young mice + control diet; YH, young mice + high-fat diet; OC, older mice + control diet; OH, older mice + high-fat diet group. n.s.: not significant.

1.2. Diet composition

The composition of the control diet (AIN-93M) and the high-fat diet (HFD-60) (Oriental Yeast, Tokyo, Japan) were as follows.

Table S1.

Nutritional Composition	Unit	AIN-93M	HFD-60
Water	g	9	9
Crude protein	g	12.74	22.02
Crude fat	g	4.35	33.21
Crude ash	g	3.05	3.06
Raw fibers	g	5.02	6.31
Soluble non-nitrogenous material	g	65.85	26.4
Total calories	kcal	353.5	492.6

Protein-calorie ratio	%	14.4	17.9
Lipid Calorie Ratio	%	11.1	60.7
Nitrogen-free extracts Calorie Ratio	%	74.5	21.4

Per 100 g of diet

1.2. Electron microscopy

Twenty random images per sample were captured using a transmission electron microscope (HT7700, Hitachi, Tokyo, Japan). The area analyzed for muscle fibers was 211,832 μm^2. The width of the target site was measured randomly at three points per image. Details of the protocol are provided in the main text.

1.3. Immunohistochemical analysis using anti-collagen VI antibody

A rabbit polyclonal anti-collagen VI antibody was used as the primary antibody (ab6588; Abcam, Cambridge, MA, USA). The immunohistochemical procedure is described in the main text. The area per image was 412,023 $\mu m^{2,}$ and three images were captured randomly using BZ51 and DP27 Microscope Digital Cameras (Olympus, Tokyo, Japan). A semi-quantitative analysis was performed to determine the intensity of collagen immunoreactivity (IR) [1]. Images were analyzed using the ImageJ Fiji software [2].

1.4. Quantitative polymerase chain reaction

The following oligonucleotide primer sets were used in polymerase chain reaction analyses:

Col6a1, 5'-GACACTCAGCGGGACACTACAC-3' (Forward) and 5'-GCGACAAAGCCAAACACATC-3' (Reverse);

B2m, 5'-TTCTGGTGCTTGTCTCACTGA-3' (Forward) and 5'- CAGTATGTTCGGCTTCCCATTC-3' (Reverse).

The messenger RNA quantitation protocol is described in the main text.

1.5. Statistical analysis

All statistical analyses are described in the main text.

2. Results

2.1. Width of muscle fibers

The widths of the muscle fibers were measured using electron microscopy (Supplementary Table. S2). There were no significant differences in muscle fiber width between the groups.

Table S2. Width of muscle fibers

	YC	YH	OC	OH
Width of muscle fiber (μm)	59.2 ± 19.3	53.9 ± 4.5	48.9 ± 2.5	50.2 ± 7.4

Data are presented as mean ± standard deviation (*n* = 3 per group). YC, young mice + control diet; YH, young mice + high-fat diet; OC, older mice + control diet; OH, older mice + high-fat diet.

2.2. Collagen VI localization

Collagen VI is a basement membrane (BM) protein component that forms a discrete network of beaded microfilaments [3]. To verify the effects of aging and obesity on BM structure, not only by collagen IV but also by other constitutive factors, we analyzed the localization of collagen VI. In all groups, the collagen IR of muscle fibers identified areas of the BM (Supplementary Fig. S2a-h). In addition, the intensity of collagen VI-IR was measured. There was an interaction between aging and high-fat diet intake, with significantly higher collagen-IR in YH than in YC (p = 0.0001) or OH (p = 0.0067) (Supplementary Fig. S2i). Furthermore, there was an interaction between aging and high-fat diet intake for the relative gene expression of *Col6a1,* a gene encoding collagen VI, with significantly higher collagen-IR in YH than in YC (p < 0.0001) and OH (p < 0.0001) (Supplementary Fig. S2j). The results suggest that a high-fat diet in youth increases collagen VI-IR and *Col6a1* expression, but such a response is unlikely to occur in old age. This result was similar to that of collagen VI, which is also a component of the BM markers.

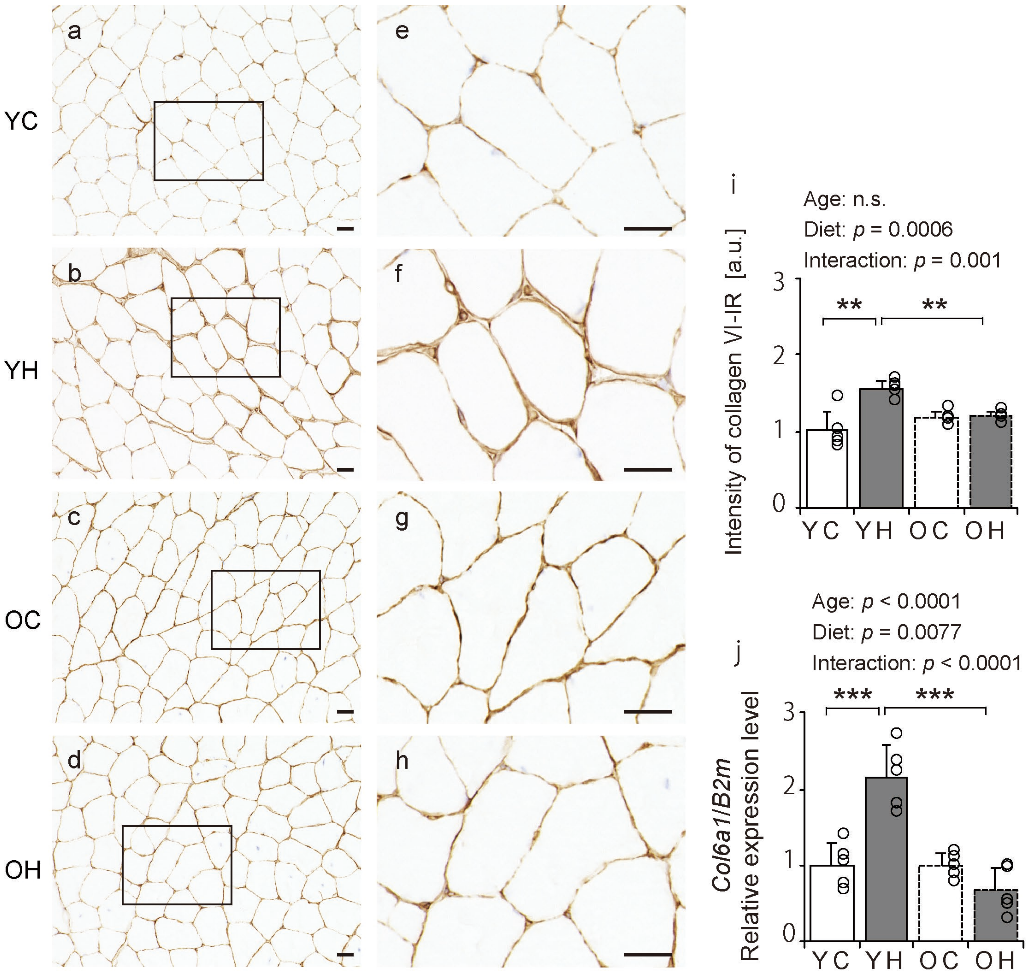

Figure S2. Comparison of collagen VI localization. Cross sections of the gastrocnemius muscle were stained by an anti-collagen VI antibody. Staining images of YC (a and e), YH (b and f), OC (c and g), and OH (d and h) are shown. e-h, are enlargements of the rectangular regions in a-d. The scale bar represents 25 μm. Collagen VI -IR intensity was measured in the images (i). Changes in relative messenger RNA expression level of *Col6a1* (j) in the YC, YH, OC, and OH groups. Data are presented as the mean ± standard deviation, n = 5 per group. YC, young mice + control diet; YH, young mice + high-fat diet; OC, older mice + control diet; OH, older mice + high-fat diet group. ***p < 0.0001, **p < 0.001. n.s.: not significant.

References

1. Crowe, A.R.; Wei, Y. Semi-quantitative determination of protein expression Using Immunohistochemistry staining and analysis: an integrated protocol. *Bio. Protoc.* **2019**, *3*, 1-11; DOI: 10.21769/BioProtoc.3465.
2. Schindelin, J.; Carreras, I.A.; Frise, E.; Kaynig, V.; Longair, M.; Pietzsch, T.; Preibisch, S.; Rueden, C.; Saalfeld, S.; Schmid, B.; Tinevez, J.Y. White, D.J.; Hartenstein, V.; Eliceiri, K.; Tomancak, P.; Cardona, A. Fiji: an open-source platform for biological-image analysis. *Nat. Methods* **2012**, *9*, 676-682; DOI: 10.1038/nmeth.2019.
3. Urciuolo, A.; Quarta, M.; Morbidoni, V.; Gattazzo, F.; Molon, S.; Grumati, P.; Montemurro, F.; Tedesco, F.S.; Blaauw, B.; Cossu, G.; Vozzi, G.; Rando, T.A.; Bonaldo, P. Collagen VI regulates satellite cell self-renewal and muscle regeneration. *Nat. Commun.* **2013**, *4*, 1964, 1-13; DOI: 10.1038/ncomms2964.
